# Supplementary material for: Functional and microstructural plasticity following social and interoceptive mental training
Source: eLife. 2023 Jul 7;12:e85188. doi: 10.7554/eLife.85188 (PMC10414971; doi:10.7554/eLife.85188)
Supplement: Supplementary file 1. — (a). Descriptive statistics Presence (b). Descriptive statistics Affect (c). Descriptive statistics Affect, excluding active controls (TC3) (d). Descriptive statistics Perspective (e). Descriptive statistics Retest controls. (f). Functional eccentricity changes GSR controlled per a-priori network. T-values and p-values below P<0.05, * indicates FDRp <0.05. (g). Functional eccentricity changes in training cohort 1 per a-priori network. T-values and p-values below P<0.05, * indicates FDRp <0.05. (h). Functional eccentricity changes in training cohort 2 per a-priori network. T-values and p-values below P<0.05, * indicates FDRp <0.05. (i). Functional eccentricity changes per a-priori network baseline to T1. T-values and p-values below P<0.05, * indicates FDRp <0.05. (j). Functional eccentricity changes per a-priori network T1 to T3. T-values and p-values below P<0.05, * indicates FDRp <0.05. (k). G1-G3 change per a-priori network Presence vs Perspective. T-values and p-values below P<0.05, * indicates FDRp <0.05. (l). G1-G3 change per a-priori network Presence vs Affect. T-values and p-values below P<0.05, * indicates FDRp <0.05. (m). G1-G3 change per a-priori network Perspective vs Affect. T-values and p-values below P<0.05, * indicates FDRp <0.05. (n). Functional eccentricity changes per a-priori network controlling for cortical thickness change. T-values and p-values below P<0.05, * indicates FDRp <0.05. (o). Functional eccentricity changes per a-priori network from baseline to T3. T-values and p-values below P<0.05, * indicates FDRp <0.05. (p). Depth-dependent qT1 change per a-priori network Training vs Retest Control. T-values and p-values below P<0.05. * indicates FDRp <0.05.(q). Descriptives of retest-control change (mean change over T0-T1; T1-T2; T2-T3) as a function of depth-dependent qT1. (r). Descriptive of Presence change (mean change over T0-T1) as a function of depth-dependent qT1 (s). Descriptives of Affect change (mean change over T0-T1, T1-T2 and T2-T3) [file elife-85188-supp1.docx]

**Functional and microstructural plasticity following social and interoceptive mental training**

**-Supplementary File 1-**

Sofie L Valk^1,2^; Philipp Kanske^3,4^; Bo-yong Park^5-7^; Seok Jun Hong^7-9^; Anne Böckler^10^; Fynn-Mathis Trautwein^11^; Boris C. Bernhardt^5^*, Tania Singer^12^*

* joint co-authors

*1. Otto Hahn Group Cognitive Neurogenetics, Max Planck Institute for Human Cognitive and Brain Sciences, Leipzig, Germany; 2. INM-7, FZ Jülich, Jülich, Germany; 3. Clinical Psychology and Behavioral Neuroscience, Faculty of Psychology, Technische Universität Dresden, Dresden, Germany; 4. Max Planck Institute for Human Cognitive and Brain Sciences, Leipzig, Germany; 5. Multimodal Imaging and Connectome Analysis Lab, McConnell Brain Imaging Centre, Montreal Neurological Institute and Hospital, McGill University, Montreal, Quebec, Canada; 6. Department of Data Science, Inha University, Incheon, South Korea; 7. Center for Neuroscience Imaging Research, Institute for Basic Science, Suwon, South Korea; 8. Center for the Developing Brain, Child Mind Institute, NY, USA; 9. Department of Biomedical Engineering, Sungkyunkwan University, Suwon, South Korea; 10. Department of Psychology, Wurzburg University, Germany; 11. Department of Psychosomatic Medicine and Psychotherapy, Medical Center – University of Freiburg, Faculty of Medicine, University of Freiburg, Freiburg im Breisgau, Germany; 12. Social Neuroscience Lab, Max Planck Society, Berlin, Germany*

**Supplementary File 1**

**Supplementary File 1a. Descriptive statistics *Presence***

| Presence | Mean | Std | CI low | CI high |
| --- | --- | --- | --- | --- |
| Attention | 0,002 | 0,013 | -0,001 | 0,004 |
| Interoception | 0,003 | 0,015 | 0,000 | 0,006 |
| Emotion | 0,001 | 0,012 | -0,002 | 0,003 |
| Empathy | 0,002 | 0,011 | 0,000 | 0,004 |
| ToM | 0,003 | 0,014 | 0,000 | 0,006 |

**Supplementary File 1b. Descriptive statistics *Affect***

| Affect | Mean | Std | CI low | CI high |
| --- | --- | --- | --- | --- |
| Attention | -0,001 | 0,012 | -0,003 | 0,001 |
| Interoception | 0,001 | 0,015 | -0,001 | 0,003 |
| Emotion | 0,001 | 0,013 | -0,001 | 0,003 |
| Empathy | 0,001 | 0,011 | -0,001 | 0,003 |
| ToM | 0,000 | 0,016 | -0,002 | 0,003 |

**Supplementary File 1c. Descriptive statistics *Affect*, excluding active controls (TC3)**

| Affect | Mean | Std | CI low | CI high |
| --- | --- | --- | --- | --- |
| Attention | -0,001 | 0,013 | -0,003 | 0,002 |
| Interoception | 0,000 | 0,015 | -0,003 | 0,003 |
| Emotion | 0,000 | 0,013 | -0,002 | 0,003 |
| Empathy | 0,000 | 0,012 | -0,002 | 0,002 |
| ToM | -0,001 | 0,016 | -0,004 | 0,002 |

**Supplementary File 1d. Descriptive statistics *Perspective***

| Perspective | Mean | Std | CI low | CI high |
| --- | --- | --- | --- | --- |
| Attention | -0,003 | 0,013 | -0,006 | -0,001 |
| Interoception | -0,003 | 0,016 | -0,006 | 0,000 |
| Emotion | 0,000 | 0,015 | -0,003 | 0,003 |
| Empathy | -0,001 | 0,012 | -0,004 | 0,001 |
| ToM | -0,001 | 0,018 | -0,005 | 0,003 |

**Supplementary File 1e. Descriptive statistics Retest controls**

| Retest controls | Mean | Std | CI low | CI high |
| --- | --- | --- | --- | --- |
| Attention | -0,001 | 0,014 | -0,003 | 0,001 |
| Interoception | 0,001 | 0,014 | -0,001 | 0,003 |
| Emotion | 0,001 | 0,013 | -0,001 | 0,002 |
| Empathy | 0,001 | 0,011 | -0,001 | 0,002 |
| ToM | 0,001 | 0,016 | -0,002 | 0,003 |

**Supplementary File 1f. Functional eccentricity changes GSR controlled per *a-priori* network.** T-values and p-values below p<0.05, ***** indicates FDRp<0.05.

|  | *Presence vs Perspective* | | *Presence vs Affect* | | *Perspective vs Affect* | |
| --- | --- | --- | --- | --- | --- | --- |
| Attention | 3,541 | 0,001* | 2,243 | 0,025 | -1,777 |  |
| Interoception | 3,203 | 0,002* | 1,552 |  | -2060 | 0,040 |
| Emotion | 1,371 |  | 0,892 |  | -0,665 |  |
| Empathy | 2,898 | 0,004* | 1,660 |  | -1,621 |  |
| ToM | 2,047 | 0,041 | 1,449 |  | -0,883 |  |

**Supplementary File 1g. Functional eccentricity changes in training cohort 1 per *a-priori* network.** T-values and p-values below p<0.05, ***** indicates FDRp<0.05.

|  | *Presence vs Perspective* | | *Presence vs Affect* | | *Perspective vs Affect* | |
| --- | --- | --- | --- | --- | --- | --- |
| Attention | 1,953 |  | 1,707 |  | -0,245 |  |
| Interoception | 2,332 | 0,021 | 0,889 |  | -1,392 |  |
| Emotion | 1,347 |  | 0,819 |  | -0,512 |  |
| Empathy | 1,984 | 0,049 | 0,950 |  | -0,100 |  |
| ToM | 1,908 |  | 1,825 |  | -0,089 |  |

**Supplementary File 1h. Functional eccentricity changes in training cohort 2 per *a-priori* network.** T-values and p-values below p<0.05, ***** indicates FDRp<0.05.

|  | *Presence vs Perspective* | | *Presence vs Affect* | | *Perspective vs Affect* | |
| --- | --- | --- | --- | --- | --- | --- |
| Attention | 2,028 | 0,044 | 0,010 |  | -1,966 |  |
| Interoception | 1,383 |  | 0,794 |  | -0,627 |  |
| Emotion | -1,016 |  | -0,623 |  | 0,421 |  |
| Empathy | 0,906 |  | 0,514 |  | -0,416 |  |
| ToM | 0,386 |  | 0,547 |  | 0,144 |  |

**Supplementary File 1i. Functional eccentricity changes per *a-priori* network baseline to T1.** T-values and p-values below p<0.05, ***** indicates FDRp<0.05.

|  | TC1-RCC | | TC2-RCC | | TC3-RCC | | TC1-TC3 | | TC2-TC3 | |
| --- | --- | --- | --- | --- | --- | --- | --- | --- | --- | --- |
| Attention | 2,529 | 0,012 | 1,523 |  | 1,144 |  | 1,424 |  | 0,440 |  |
| Interoception | -0,260 |  | -0,813 |  | -0,756 |  | 0,505 |  | -0,093 |  |
| Emotion | 0,411 |  | -1,059 |  | 0,492 |  | -0,077 |  | -1,553 |  |
| Empathy | 0,608 |  | 0,039 |  | 0,348 |  | 0,269 |  | -0,296 |  |
| ToM | 1,225 |  | 0,358 |  | 0,763 |  | 0,476 |  | -0,379 |  |

**Supplementary File 1j. Functional eccentricity changes per *a-priori* network T1 to T3.** T-values and p-values below p<0.05, ***** indicates FDRp<0.05.

|  | *Perspective* vs Retest Control | | *Affect* vs Retest Control | | *Perspective-Affect* | |
| --- | --- | --- | --- | --- | --- | --- |
| Attention | -1,981 | 0,048 | -0,431 |  | -1,528 |  |
| Interoception | -0,960 |  | 0,544 |  | -1,461 |  |
| Emotion | -0,143 |  | -0,045 |  | -0,097 |  |
| Empathy | -1,192 |  | -0,113 |  | -1,059 |  |
| ToM | -0,818 |  | -0,858 |  | 0,022 |  |

**Supplementary File 1k. G1-G3 change per *a-priori* network *Presence* vs *Perspective*.** T-values and p-values below p<0.05, ***** indicates FDRp<0.05.

|  | G1 |  | G2 |  | G3 |  |
| --- | --- | --- | --- | --- | --- | --- |
| Attention | -1,321 |  | 2,508 | 0.012 | 1,098 |  |
| Interoception | -1,787 |  | -2,180 | 0.030 | -1,499 |  |
| Emotion | -0,118 |  | 1,501 |  | 0,833 |  |
| Empathy | -0,573 |  | 1,713 |  | -0,206 |  |
| ToM | 1,048 |  | 2,102 | 0.036 | -0,947 |  |

**Supplementary File 1l. G1-G3 change per *a-priori* network *Presence* vs *Affect*.** T-values and p-values below p<0.05, ***** indicates FDRp<0.05.

|  | G1 |  | G2 |  | G3 |  |
| --- | --- | --- | --- | --- | --- | --- |
| Attention | -0.945 |  | 1.715 |  | 0,113 |  |
| Interoception | -2.185 | 0.03 | -0.901 |  | -1,339 |  |
| Emotion | -0.643 |  | 0.871 |  | -0,782 |  |
| Empathy | -0.976 |  | 3.215 | 0.002* | -1,824 |  |
| ToM | 1.135 |  | 2.382 | 0.018 | -0,925 |  |

**Supplementary File 1m. G1-G3 change per *a-priori* network *Perspective* vs *Affect*.** T-values and p-values below p<0.05, ***** indicates FDRp<0.05.

|  | G1 |  | G2 |  | G3 |  |
| --- | --- | --- | --- | --- | --- | --- |
| Attention | 0.527 |  | -1.081 |  | -1,087 |  |
| Interoception | -0.156 |  | 1.506 |  | 0,344 |  |
| Emotion | -0.490 |  | -0.796 |  | -1,654 |  |
| Empathy | -0.315 |  | 1.228 |  | -1,531 |  |
| ToM | -0.050 |  | 0.002 |  | 0,140 |  |

**Supplementary File 1n. Functional eccentricity changes per *a-priori* network controlling for cortical thickness change.** T-values and p-values below p<0.05, ***** indicates FDRp<0.05.

|  | *Presence vs Perspective* | | *Presence vs Affect* | | *Perspective vs Affect* | |
| --- | --- | --- | --- | --- | --- | --- |
| Attention | 2,859 | 0,005* | 1,479 |  | -1,691 |  |
| Interoception | 2,744 | 0,007* | 1,064 |  | -1,964 | 0.050 |
| Emotion | 0,374 |  | -0,165 |  | -0,566 |  |
| Empathy | 2,206 | 0,028 | 0,870 |  | -1,566 |  |
| ToM | 1,709 |  | 1,316 |  | -0,595 |  |

**Supplementary File 1o. Functional eccentricity changes per *a-priori* network from baseline to T3.** T-values and p-values below p<0.05, ***** indicates FDRp<0.05.

|  | Training vs Retest Control |  |
| --- | --- | --- |
| Attention | -0,567 |  |
| Interoception | -0,380 |  |
| Emotion | -0,299 |  |
| Empathy | -0,651 |  |
| ToM | -0,192 |  |

**Supplementary File 1p. Depth-dependent qT1 change per *a-priori* network Training vs Retest Control.** T-values and p-values below p<0.05. ***** indicates FDRp<0.05.

| Depth | Attention | | Interoception | | Emotion |  | Empathy | | ToM |  |
| --- | --- | --- | --- | --- | --- | --- | --- | --- | --- | --- |
| 1 | -1,368 |  | -0,190 |  | -0,414 |  | -1,099 |  | -0,799 |  |
| 2 | -2,012 |  | -0,454 |  | -0,669 |  | -1,451 |  | -1,338 |  |
| 3 | -2,590 | 0,011 | -0,865 |  | -1,013 |  | -1,807 |  | -1,822 |  |
| 4 | -3,080 | 0,002* | -1,295 |  | -1,441 |  | -2,152 | 0,033* | -2,242 | 0,026* |
| 5 | -3,493 | 0,001* | -1,733 |  | -1,923 |  | -2,531 | 0,012* | -2,617 | 0,010* |
| 6 | -3,820 | 0,001* | -2,164 | 0,032* | -2,367 | 0,019* | -2,958 | 0,004* | -2,976 | 0,003* |
| 7 | -4,035 | 0,001* | -2,512 | 0,013* | -2,615 | 0,010* | -3,340 | 0,001* | -3,249 | 0,001* |
| 8 | -4,090 | 0,001* | -2,754 | 0,007* | -2,622 | 0,010* | -3,523 | 0,001* | -3,262 | 0,001* |
| 9 | -3,987 | 0,001* | -2,859 | 0,005* | -2,481 | 0,014* | -3,497 | 0,001* | -3,023 | 0,003* |
| 10 | -3,819 | 0,001* | -2,769 | 0,006* | -2,360 | 0,020* | -3,388 | 0,001* | -2,733 | 0,007* |
| 11 | -3,694 | 0,001* | -2,551 | 0,012* | -2,324 | 0,021* | -3,303 | 0,001* | -2,482 | 0,014* |
| 12 | -3,659 | 0,001* | -2,288 | 0,023* | -2,363 | 0,019* | -3,256 | 0,001* | -2,282 | 0,024* |

**Supplementary File 1q. Descriptives of retest-control change (mean change over T0-T1; T1-T2; T2-T3) as a function of depth-dependent qT1**

|  | Attention | | | | Interoception | | | | Emotion | | | |
| --- | --- | --- | --- | --- | --- | --- | --- | --- | --- | --- | --- | --- |
| Depth | Mean | Std | CI min | CI  max | Mean | Std | CI  min | CI  max | Mean | Std | CI  min | CI  max |
| 1 | 7,562 | 49,980 | -0,050 | 15,175 | 1,997 | 32,385 | -2,936 | 6,929 | -2,210 | 28,341 | -6,527 | 2,107 |
| 2 | 7,081 | 40,984 | 0,838 | 13,323 | 1,862 | 25,168 | -1,971 | 5,696 | -2,224 | 21,211 | -5,455 | 1,007 |
| 3 | 6,366 | 34,091 | 1,173 | 11,559 | 1,737 | 20,467 | -1,381 | 4,854 | -2,101 | 16,432 | -4,604 | 0,402 |
| 4 | 5,510 | 28,803 | 1,123 | 9,897 | 1,535 | 17,499 | -1,130 | 4,201 | -1,910 | 13,395 | -3,950 | 0,130 |
| 5 | 4,594 | 24,717 | 0,829 | 8,359 | 1,267 | 15,458 | -1,087 | 3,622 | -1,647 | 11,497 | -3,399 | 0,104 |
| 6 | 3,718 | 21,721 | 0,409 | 7,026 | 0,933 | 13,867 | -1,180 | 3,045 | -1,350 | 10,542 | -2,955 | 0,256 |
| 7 | 2,951 | 19,821 | -0,069 | 5,970 | 0,546 | 12,560 | -1,367 | 2,460 | -1,069 | 10,405 | -2,654 | 0,516 |
| 8 | 2,357 | 19,003 | -0,537 | 5,252 | 0,159 | 11,639 | -1,614 | 1,932 | -0,844 | 10,843 | -2,495 | 0,808 |
| 9 | 1,987 | 19,123 | -0,925 | 4,900 | -0,199 | 11,126 | -1,894 | 1,496 | -0,697 | 11,507 | -2,450 | 1,056 |
| 10 | 1,841 | 19,858 | -1,184 | 4,865 | -0,529 | 10,911 | -2,191 | 1,133 | -0,630 | 12,042 | -2,464 | 1,204 |
| 11 | 1,891 | 20,844 | -1,284 | 5,066 | -0,831 | 10,900 | -2,491 | 0,829 | -0,670 | 12,163 | -2,522 | 1,183 |
| 12 | 2,088 | 21,799 | -1,232 | 5,409 | -1,073 | 10,979 | -2,745 | 0,600 | -0,814 | 11,806 | -2,612 | 0,984 |
|  | **Empathy** | | | | **ToM** | | | |  |  |  |  |
| Depth | Mean | Std | CI min | CI  max | Mean | Std | CI min | CI  max |  |  |  |  |
| 1 | 2,651 | 30,526 | -1,999 | 7,301 | 1,606 | 25,823 | -2,328 | 5,539 |  |  |  |  |
| 2 | 1,865 | 23,063 | -1,648 | 5,378 | 1,080 | 19,190 | -1,844 | 4,003 |  |  |  |  |
| 3 | 1,270 | 17,727 | -1,430 | 3,970 | 0,669 | 14,902 | -1,601 | 2,938 |  |  |  |  |
| 4 | 0,828 | 14,162 | -1,329 | 2,985 | 0,383 | 12,308 | -1,492 | 2,258 |  |  |  |  |
| 5 | 0,531 | 11,871 | -1,278 | 2,339 | 0,207 | 10,757 | -1,431 | 1,846 |  |  |  |  |
| 6 | 0,343 | 10,550 | -1,264 | 1,950 | 0,121 | 9,905 | -1,388 | 1,629 |  |  |  |  |
| 7 | 0,237 | 10,039 | -1,292 | 1,766 | 0,090 | 9,681 | -1,385 | 1,565 |  |  |  |  |
| 8 | 0,171 | 10,209 | -1,384 | 1,726 | 0,077 | 10,020 | -1,449 | 1,604 |  |  |  |  |
| 9 | 0,123 | 10,749 | -1,514 | 1,761 | 0,057 | 10,617 | -1,560 | 1,674 |  |  |  |  |
| 10 | 0,061 | 11,323 | -1,664 | 1,786 | -0,001 | 11,112 | -1,694 | 1,692 |  |  |  |  |
| 11 | -0,034 | 11,723 | -1,819 | 1,752 | -0,112 | 11,273 | -1,829 | 1,605 |  |  |  |  |
| 12 | -0,160 | 11,866 | -1,967 | 1,647 | -0,274 | 11,079 | -1,962 | 1,413 |  |  |  |  |

**Supplementary File 1r. Descriptive of *Presence* change (mean change over T0-T1) as a function of depth-dependent qT1**

|  | Attention | | | | | | | Interoception | | | | | | | Emotion | | | | |
| --- | --- | --- | --- | --- | --- | --- | --- | --- | --- | --- | --- | --- | --- | --- | --- | --- | --- | --- | --- |
| Depth | Mean | Std | CI  min | | | CI  max | | Mean | | Std | | CI  min | | CI  max | Mean | | Std | | |
| 1 | 26,962 | 42,445 | 18,904 | | | 35,021 | | 11,744 | | 32,692 | | 5,5366 | | 17,95 | -0,1614 | | 29,164 | | |
| 2 | 22,432 | 37,77 | 15,261 | | | 29,602 | | 10,628 | | 24,888 | | 5,9029 | | 15,35 | -0,75176 | | 21,216 | | |
| 3 | 17,804 | 34,339 | 11,284 | | | 24,324 | | 9,0319 | | 20,491 | | 5,1414 | | 12,92 | -1,2386 | | 17,084 | | |
| 4 | 13,436 | 31,073 | 7,5364 | | | 19,335 | | 7,2546 | | 18,145 | | 3,8096 | | 10,7 | -1,5975 | | 14,796 | | |
| 5 | 9,636 | 27,622 | 4,3918 | | | 14,88 | | 5,4632 | | 16,527 | | 2,3254 | | 8,601 | -1,8056 | | 13,27 | | |
| 6 | 6,6193 | 24,229 | 2,0193 | | | 11,219 | | 3,7751 | | 15,091 | | 0,90993 | | 6,64 | -1,8841 | | 12,369 | | |
| 7 | 4,4032 | 21,348 | 0,35014 | | | 8,4563 | | 2,3068 | | 13,884 | | -0,32914 | | 4,943 | -1,8585 | | 12,251 | | |
| 8 | 2,9186 | 19,435 | -0,77129 | | | 6,6085 | | 1,0881 | | 13,073 | | -1,3938 | | 3,57 | -1,8242 | | 12,83 | | |
| 9 | 2,048 | 18,648 | -1,4924 | | | 5,5884 | | 0,09958 | | 12,75 | | -2,3212 | | 2,52 | -1,8667 | | 13,723 | | |
| 10 | 1,6593 | 18,743 | -1,8993 | | | 5,2179 | | -0,69095 | | 12,736 | | -3,1091 | | 1,727 | -2,0169 | | 14,379 | | |
| 11 | 1,5937 | 19,274 | -2,0655 | | | 5,253 | | -1,3077 | | 12,618 | | -3,7032 | | 1,088 | -2,2674 | | 14,323 | | |
| 12 | 1,732 | 19,852 | -2,0371 | | | 5,5011 | | -1,7709 | | 12,094 | | -4,0669 | | 0,525 | -2,5839 | | 13,256 | | |
|  |  |  | | **Empathy** | | | | | | | **ToM** | | | | | | | |  |
| Depth | CI  min | CI  max | | Mean | Std | | CI min | | CI  max | | Mean | | Std | | | CI min | | CI  max |  |
| 1 | -5,6984 | 5,3756 | | 10,435 | 28,632 | | 4,9986 | | 15,87 | | 8,5313 | | 22,468 | | | 4,2655 | | 12,8 |  |
| 2 | -4,7798 | 3,2763 | | 8,126 | 22,669 | | 3,8221 | | 12,43 | | 5,8231 | | 16,579 | | | 2,6755 | | 8,971 |  |
| 3 | -4,4821 | 2,0049 | | 6,1404 | 19,553 | | 2,4281 | | 9,853 | | 3,7308 | | 14,063 | | | 1,0608 | | 6,401 |  |
| 4 | -4,4067 | 1,2117 | | 4,506 | 17,422 | | 1,1984 | | 7,814 | | 2,1832 | | 13,019 | | | -0,28858 | | 4,655 |  |
| 5 | -4,3251 | 0,71385 | | 3,2537 | 15,371 | | 0,33528 | | 6,172 | | 1,1235 | | 12,101 | | | -1,1739 | | 3,421 |  |
| 6 | -4,2325 | 0,46425 | | 2,39 | 13,492 | | -0,17144 | | 4,952 | | 0,53595 | | 11,18 | | | -1,5866 | | 2,659 |  |
| 7 | -4,1844 | 0,46747 | | 1,7933 | 12,311 | | -0,54403 | | 4,131 | | 0,30078 | | 10,795 | | | -1,7487 | | 2,35 |  |
| 8 | -4,2601 | 0,61178 | | 1,3569 | 12,164 | | -0,95258 | | 3,666 | | 0,26041 | | 11,319 | | | -1,8886 | | 2,409 |  |
| 9 | -4,4722 | 0,73882 | | 0,9688 | 12,865 | | -1,4737 | | 3,411 | | 0,27816 | | 12,455 | | | -2,0866 | | 2,643 |  |
| 10 | -4,7468 | 0,71295 | | 0,591 | 13,757 | | -2,0208 | | 3,203 | | 0,25582 | | 13,511 | | | -2,3094 | | 2,821 |  |
| 11 | -4,9868 | 0,45196 | | 0,2205 | 14,239 | | -2,4828 | | 2,924 | | 0,12787 | | 13,9 | | | -2,5111 | | 2,767 |  |
| 12 | -5,1007 | -0,06723 | | -0,136 | 14,066 | | -2,8062 | | 2,535 | | -0,1157 | | 13,375 | | | -2,655 | | 2,424 |  |

**Supplementary File 1s. Descriptives of *Affect* change (mean change over T0-T1, T1-T2 and T2-T3) as a function of depth-dependent qT1**

|  | Attention | | | | | | | Interoception | | | | | | | Emotion | | | |
| --- | --- | --- | --- | --- | --- | --- | --- | --- | --- | --- | --- | --- | --- | --- | --- | --- | --- | --- |
| Depth | Mean | Std | CI  min | | | CI  max | | Mean | | Std | | CI  min | | CI  max | Mean | | Std | |
| 1 | 4,706 | 44,870 | -2,212 | | | 11,625 | | 1,224 | | 29,272 | | -3,289 | | 5,738 | -6,308 | | 25,848 | |
| 2 | 2,206 | 38,832 | -3,782 | | | 8,193 | | 0,772 | | 23,371 | | -2,832 | | 4,376 | -5,746 | | 19,466 | |
| 3 | 0,288 | 33,868 | -4,934 | | | 5,510 | | 0,107 | | 19,716 | | -2,933 | | 3,147 | -5,139 | | 15,474 | |
| 4 | -1,147 | 29,495 | -5,695 | | | 3,401 | | -0,647 | | 17,393 | | -3,329 | | 2,035 | -4,647 | | 12,938 | |
| 5 | -2,203 | 25,537 | -6,140 | | | 1,735 | | -1,357 | | 15,627 | | -3,767 | | 1,053 | -4,306 | | 11,271 | |
| 6 | -2,943 | 22,076 | -6,347 | | | 0,461 | | -1,969 | | 14,056 | | -4,137 | | 0,198 | -4,102 | | 10,373 | |
| 7 | -3,446 | 19,395 | -6,437 | | | -0,456 | | -2,498 | | 12,669 | | -4,451 | | -0,544 | -4,024 | | 10,246 | |
| 8 | -3,791 | 17,745 | -6,528 | | | -1,055 | | -2,982 | | 11,649 | | -4,778 | | -1,186 | -4,065 | | 10,788 | |
| 9 | -4,057 | 17,136 | -6,700 | | | -1,415 | | -3,421 | | 11,112 | | -5,134 | | -1,707 | -4,187 | | 11,688 | |
| 10 | -4,303 | 17,292 | -6,969 | | | -1,637 | | -3,799 | | 10,982 | | -5,493 | | -2,106 | -4,350 | | 12,497 | |
| 11 | -4,529 | 17,818 | -7,276 | | | -1,781 | | -4,111 | | 11,001 | | -5,807 | | -2,415 | -4,471 | | 12,750 | |
| 12 | -4,699 | 18,383 | -7,534 | | | -1,865 | | -4,300 | | 10,904 | | -5,982 | | -2,619 | -4,496 | | 12,191 | |
|  |  |  | | **Empathy** | | | | | | | **ToM** | | | | | | | |
| Depth | CI  min | CI  max | | Mean | Std | | CI min | | CI  max | | Mean | | Std | | | CI min | | CI  max |
| 1 | -10,293 | -2,322 | | -0,781 | 26,852 | | -4,921 | | 3,359 | | -0,289 | | 20,940 | | | -3,517 | | 2,940 |
| 2 | -8,748 | -2,745 | | -1,624 | 21,451 | | -4,931 | | 1,684 | | -1,155 | | 17,108 | | | -3,793 | | 1,483 |
| 3 | -7,525 | -2,753 | | -2,234 | 17,936 | | -5,000 | | 0,531 | | -1,769 | | 14,871 | | | -4,062 | | 0,524 |
| 4 | -6,642 | -2,652 | | -2,629 | 15,423 | | -5,007 | | -0,251 | | -2,190 | | 13,319 | | | -4,243 | | -0,136 |
| 5 | -6,044 | -2,568 | | -2,889 | 13,394 | | -4,954 | | -0,824 | | -2,449 | | 12,020 | | | -4,303 | | -0,596 |
| 6 | -5,702 | -2,503 | | -3,073 | 11,807 | | -4,894 | | -1,253 | | -2,576 | | 11,004 | | | -4,273 | | -0,880 |
| 7 | -5,604 | -2,445 | | -3,224 | 10,873 | | -4,901 | | -1,548 | | -2,618 | | 10,536 | | | -4,242 | | -0,993 |
| 8 | -5,729 | -2,402 | | -3,366 | 10,721 | | -5,019 | | -1,713 | | -2,621 | | 10,736 | | | -4,276 | | -0,965 |
| 9 | -5,989 | -2,385 | | -3,529 | 11,195 | | -5,255 | | -1,803 | | -2,625 | | 11,397 | | | -4,383 | | -0,868 |
| 10 | -6,277 | -2,423 | | -3,714 | 11,910 | | -5,550 | | -1,877 | | -2,659 | | 12,160 | | | -4,534 | | -0,784 |
| 11 | -6,437 | -2,505 | | -3,911 | 12,458 | | -5,832 | | -1,990 | | -2,731 | | 12,680 | | | -4,686 | | -0,776 |
| 12 | -6,376 | -2,617 | | -4,114 | 12,614 | | -6,059 | | -2,169 | | -2,843 | | 12,745 | | | -4,809 | | -0,878 |

**Supplementary File 1t. Descriptives of *Affect* change (mean change over T1-T2 and T2-T3) as a function of depth-dependent qT1**

|  | Attention | | | | | | | Interoception | | | | | | | Emotion | | | | |
| --- | --- | --- | --- | --- | --- | --- | --- | --- | --- | --- | --- | --- | --- | --- | --- | --- | --- | --- | --- |
| Depth | Mean | Std | CI  min | | | CI  max | | Mean | | Std | | CI  min | | CI  max | Mean | | Std | | |
| 1 | -2,5228 | 46,28 | -11,523 | | | 6,4775 | | -1,6892 | | 30,662 | | -7,6521 | | 4,274 | -5,2022 | | 26,077 | | |
| 2 | -4,0713 | 39,93 | -11,837 | | | 3,6942 | | -2,287 | | 24,126 | | -6,9789 | | 2,405 | -4,9854 | | 19,189 | | |
| 3 | -4,9563 | 34,624 | -11,69 | | | 1,7773 | | -2,8899 | | 19,931 | | -6,766 | | 0,986 | -4,6893 | | 14,886 | | |
| 4 | -5,3472 | 29,986 | -11,179 | | | 0,4843 | | -3,416 | | 17,237 | | -6,7682 | | -0,064 | -4,4435 | | 12,235 | | |
| 5 | -5,4408 | 25,904 | -10,478 | | | -0,4032 | | -3,7642 | | 15,357 | | -6,7508 | | -0,778 | -4,2724 | | 10,623 | | |
| 6 | -5,3062 | 22,483 | -9,6786 | | | -0,9339 | | -3,9564 | | 13,899 | | -6,6593 | | -1,254 | -4,1187 | | 9,9775 | | |
| 7 | -5,063 | 19,975 | -8,9476 | | | -1,1785 | | -4,0454 | | 12,751 | | -6,5251 | | -1,566 | -4,0143 | | 10,208 | | |
| 8 | -4,7844 | 18,592 | -8,4001 | | | -1,1687 | | -4,1359 | | 11,948 | | -6,4596 | | -1,812 | -3,9849 | | 11,065 | | |
| 9 | -4,5494 | 18,277 | -8,1038 | | | -0,995 | | -4,2382 | | 11,532 | | -6,4808 | | -1,996 | -3,9986 | | 12,15 | | |
| 10 | -4,4092 | 18,688 | -8,0435 | | | -0,7749 | | -4,3449 | | 11,422 | | -6,5663 | | -2,124 | -4,0351 | | 12,983 | | |
| 11 | -4,3492 | 19,414 | -8,1248 | | | -0,5736 | | -4,4493 | | 11,388 | | -6,664 | | -2,235 | -4,0192 | | 13,092 | | |
| 12 | -4,3433 | 20,116 | -8,2554 | | | -0,4312 | | -4,49 | | 11,182 | | -6,6646 | | -2,316 | -3,8985 | | 12,234 | | |
|  |  |  | | **Empathy** | | | | | | | **ToM** | | | | | | | |  |
| Depth | CI  min | CI  max | | Mean | Std | | CI min | | CI  max | | Mean | | Std | | | CI min | | CI  max |  |
| 1 | -10,273 | -0,13092 | | -3,236 | 27,882 | | -8,6579 | | 2,187 | | -2,019 | | 20,485 | | | -6,0027 | | 1,965 |  |
| 2 | -8,7171 | -1,2537 | | -4,01 | 22,161 | | -8,3195 | | 0,3 | | -2,7088 | | 16,61 | | | -5,9391 | | 0,522 |  |
| 3 | -7,5841 | -1,7944 | | -4,491 | 18,239 | | -8,0381 | | -0,944 | | -3,1489 | | 14,324 | | | -5,9345 | | -0,363 |  |
| 4 | -6,8228 | -2,0641 | | -4,685 | 15,376 | | -7,6756 | | -1,695 | | -3,4087 | | 12,741 | | | -5,8864 | | -0,931 |  |
| 5 | -6,3384 | -2,2065 | | -4,688 | 13,145 | | -7,2448 | | -2,132 | | -3,5069 | | 11,45 | | | -5,7338 | | -1,28 |  |
| 6 | -6,0591 | -2,1783 | | -4,599 | 11,56 | | -6,8469 | | -2,351 | | -3,4807 | | 10,514 | | | -5,5254 | | -1,436 |  |
| 7 | -5,9995 | -2,029 | | -4,473 | 10,79 | | -6,5717 | | -2,375 | | -3,3644 | | 10,184 | | | -5,3449 | | -1,384 |  |
| 8 | -6,1367 | -1,8331 | | -4,35 | 10,863 | | -6,4623 | | -2,237 | | -3,2093 | | 10,526 | | | -5,2563 | | -1,162 |  |
| 9 | -6,3615 | -1,6357 | | -4,263 | 11,539 | | -6,5068 | | -2,019 | | -3,0571 | | 11,297 | | | -5,254 | | -0,86 |  |
| 10 | -6,56 | -1,5102 | | -4,233 | 12,413 | | -6,6467 | | -1,819 | | -2,932 | | 12,161 | | | -5,297 | | -0,567 |  |
| 11 | -6,5652 | -1,4731 | | -4,244 | 13,099 | | -6,7913 | | -1,696 | | -2,8149 | | 12,79 | | | -5,3022 | | -0,328 |  |
| 12 | -6,2778 | -1,5192 | | -4,286 | 13,409 | | -6,8935 | | -1,678 | | -2,7091 | | 12,996 | | | -5,2365 | | -0,182 |  |

**Supplementary File 1u. Descriptives of *Perspective* change (mean change over T1-T2 and T2-T3) as a function of depth-dependent qT1**

|  | Attention | | | | | | | Interoception | | | | | | | Emotion | | | | |
| --- | --- | --- | --- | --- | --- | --- | --- | --- | --- | --- | --- | --- | --- | --- | --- | --- | --- | --- | --- |
| Depth | Mean | Std | CI  min | | | CI  max | | Mean | | Std | | CI  min | | CI  max | Mean | | Std | | |
| 1 | -10,87 | 52,435 | -21,494 | | | -0,2458 | | -2,7462 | | 34,112 | | -9,6579 | | 4,165 | -2,7201 | | 27,766 | | |
| 2 | -9,2606 | 44,418 | -18,26 | | | -0,2608 | | -3,2496 | | 27,916 | | -8,9059 | | 2,407 | -2,9624 | | 21,214 | | |
| 3 | -7,5768 | 38,091 | -15,295 | | | 0,1412 | | -3,371 | | 23,751 | | -8,1835 | | 1,442 | -2,6939 | | 17,138 | | |
| 4 | -5,9606 | 32,896 | -12,626 | | | 0,7047 | | -3,2017 | | 20,677 | | -7,3913 | | 0,988 | -2,237 | | 14,413 | | |
| 5 | -4,5253 | 28,585 | -10,317 | | | 1,2667 | | -2,8753 | | 18,163 | | -6,5554 | | 0,805 | -1,7131 | | 12,627 | | |
| 6 | -3,3834 | 25,12 | -8,4732 | | | 1,7064 | | -2,4586 | | 16,021 | | -5,7048 | | 0,788 | -1,2131 | | 11,73 | | |
| 7 | -2,534 | 22,66 | -7,1253 | | | 2,0573 | | -1,9479 | | 14,33 | | -4,8514 | | 0,956 | -0,74685 | | 11,697 | | |
| 8 | -1,947 | 21,333 | -6,2694 | | | 2,3755 | | -1,3666 | | 13,157 | | -4,0326 | | 1,299 | -0,35126 | | 12,308 | | |
| 9 | -1,58 | 21,038 | -5,8427 | | | 2,6827 | | -0,79743 | | 12,511 | | -3,3324 | | 1,738 | -0,045665 | | 13,176 | | |
| 10 | -1,3974 | 21,448 | -5,7432 | | | 2,9483 | | -0,28119 | | 12,209 | | -2,7549 | | 2,193 | 0,17334 | | 13,835 | | |
| 11 | -1,3603 | 22,168 | -5,8519 | | | 3,1313 | | 0,14272 | | 12,018 | | -2,2924 | | 2,578 | 0,21532 | | 13,883 | | |
| 12 | -1,4199 | 22,878 | -6,0554 | | | 3,2156 | | 0,45214 | | 11,769 | | -1,9325 | | 2,837 | 0,083819 | | 13,132 | | |
|  |  |  | | **Empathy** | | | | | | | **ToM** | | | | | | | |  |
| depth | CI  min | CI  max | | Mean | Std | | CI min | | CI  max | | Mean | | Std | | | CI min | | CI  max |  |
| 1 | -8,3461 | 2,9059 | | -4,22 | 32,346 | | -10,774 | | 2,334 | | -4,5243 | | 23,152 | | | -9,2154 | | 0,167 |  |
| 2 | -7,2608 | 1,336 | | -3,914 | 25,651 | | -9,1112 | | 1,284 | | -3,8974 | | 18,304 | | | -7,6062 | | -0,189 |  |
| 3 | -6,1663 | 0,77853 | | -3,437 | 20,779 | | -7,6477 | | 0,773 | | -3,1972 | | 15,084 | | | -6,2534 | | -0,141 |  |
| 4 | -5,1574 | 0,68333 | | -2,87 | 17,117 | | -6,3379 | | 0,599 | | -2,5082 | | 12,903 | | | -5,1227 | | 0,106 |  |
| 5 | -4,2715 | 0,84524 | | -2,307 | 14,48 | | -5,2405 | | 0,627 | | -1,9118 | | 11,426 | | | -4,227 | | 0,403 |  |
| 6 | -3,5899 | 1,1637 | | -1,828 | 12,794 | | -4,4202 | | 0,765 | | -1,443 | | 10,636 | | | -3,598 | | 0,712 |  |
| 7 | -3,1169 | 1,6232 | | -1,422 | 12,087 | | -3,8706 | | 1,027 | | -1,1308 | | 10,646 | | | -3,2879 | | 1,026 |  |
| 8 | -2,8451 | 2,1426 | | -1,113 | 12,185 | | -3,5823 | | 1,356 | | -0,94722 | | 11,328 | | | -3,2424 | | 1,348 |  |
| 9 | -2,7153 | 2,624 | | -0,893 | 12,736 | | -3,474 | | 1,687 | | -0,83875 | | 12,296 | | | -3,3301 | | 1,653 |  |
| 10 | -2,6299 | 2,9766 | | -0,758 | 13,363 | | -3,4655 | | 1,95 | | -0,79372 | | 13,216 | | | -3,4715 | | 1,884 |  |
| 11 | -2,5977 | 3,0283 | | -0,722 | 13,757 | | -3,5091 | | 2,066 | | -0,81567 | | 13,77 | | | -3,6058 | | 1,975 |  |
| 12 | -2,577 | 2,7447 | | -0,759 | 13,798 | | -3,5551 | | 2,036 | | -0,89601 | | 13,851 | | | -3,7024 | | 1,91 |  |

**Supplementary File 1v. Depth-dependent qT1 change per *a-priori* network *Presence* vs *Perspective*.** T-values and p-values below p<0.05, and Cohen’s D effect size, ***** indicates FDRp<0.05.

|  | Attention | | | Interoception | | | Emotion | | | Empathy | | | ToM | | |
| --- | --- | --- | --- | --- | --- | --- | --- | --- | --- | --- | --- | --- | --- | --- | --- |
| depth | t | p | D | t- | p | D | t | p | D | t | p | D | t | p | D |
| 1 | 5,663 | 0,001* | 0,491 | 3,217 | 0,001* | 0,279 | 0,639 |  | 0,055 | 3,539 | 0,001* | 0,307 | 3,992 | 0,001* | 0,346 |
| 2 | 5,581 | 0,001* | 0,484 | 3,914 | 0,001* | 0,340 | 0,753 |  | 0,065 | 3,725 | 0,001* | 0,323 | 3,868 | 0,001* | 0,336 |
| 3 | 5,177 | 0,001* | 0,449 | 4,207 | 0,001* | 0,365 | 0,630 |  | 0,055 | 3,642 | 0,001* | 0,316 | 3,349 | 0,001* | 0,291 |
| 4 | 4,552 | 0,001* | 0,395 | 4,065 | 0,001* | 0,353 | 0,332 |  | 0,029 | 3,330 | 0,001* | 0,289 | 2,604 | 0,009* | 0,226 |
| 5 | 3,816 | 0,001* | 0,331 | 3,629 | 0,001* | 0,315 | -0,056 |  | -0,005 | 2,920 | 0,004* | 0,253 | 1,878 |  | 0,163 |
| 6 | 3,075 | 0,002* | 0,267 | 3,014 | 0,003* | 0,262 | -0,434 |  | -0,038 | 2,508 | 0,012* | 0,218 | 1,324 |  | 0,115 |
| 7 | 2,376 | 0,018* | 0,206 | 2,262 | 0,024 | 0,196 | -0,729 |  | -0,063 | 2,042 | 0,042 | 0,177 | 0,977 |  | 0,085 |
| 8 | 1,778 |  | 0,154 | 1,394 |  | 0,121 | -0,934 |  | -0,081 | 1,560 |  | 0,135 | 0,786 |  | 0,068 |
| 9 | 1,344 |  | 0,117 | 0,511 |  | 0,044 | -1,085 |  | -0,094 | 1,109 |  | 0,096 | 0,673 |  | 0,058 |
| 10 | 1,109 |  | 0,096 | -0,280 |  | -0,024 | -1,240 |  | -0,108 | 0,749 |  | 0,065 | 0,591 |  | 0,051 |
| 11 | 1,037 |  | 0,090 | -0,916 |  | -0,080 | -1,394 |  | -0,121 | 0,495 |  | 0,043 | 0,515 |  | 0,045 |
| 12 | 1,075 |  | 0,093 | -1,414 |  | -0,123 | -1,570 |  | -0,136 | 0,321 |  | 0,028 | 0,434 |  | 0,038 |

**Supplementary File 1w. Depth-dependent qT1 change per *a-priori* network *Presence* vs *Affect*.** T-values and p-values below p<0.05, and Cohen’s D effect size, ***** indicates FDRp<0.05.

|  | Attention | | | Interoception | | | Emotion | | | Empathy | | | ToM | | |
| --- | --- | --- | --- | --- | --- | --- | --- | --- | --- | --- | --- | --- | --- | --- | --- |
| Depth | t | p | D | t- | p | D | t | p | D | t | p | D | t | p | D |
| 1 | 3,840 | 0,001* | 0,333 | 2,722 | 0,007* | 0,236 | 1,800 |  | 0,156 | 3,082 | 0,002* | 0,267 | 3,081 | 0,002* | 0,267 |
| 2 | 4,094 | 0,001* | 0,355 | 3,243 | 0,001* | 0,281 | 1,940 |  | 0,168 | 3,419 | 0,001* | 0,297 | 3,167 | 0,002* | 0,275 |
| 3 | 4,101 | 0,001* | 0,356 | 3,536 | 0,000* | 0,307 | 1,914 |  | 0,166 | 3,592 | 0,001* | 0,312 | 3,019 | 0,003* | 0,262 |
| 4 | 3,928 | 0,001* | 0,341 | 3,591 | 0,000* | 0,312 | 1,793 |  | 0,156 | 3,640 | 0,001* | 0,316 | 2,750 | 0,006* | 0,239 |
| 5 | 3,671 | 0,001* | 0,319 | 3,478 | 0,001* | 0,302 | 1,680 |  | 0,146 | 3,647 | 0,001* | 0,317 | 2,504 | 0,013* | 0,217 |
| 6 | 3,396 | 0,001* | 0,295 | 3,266 | 0,001* | 0,283 | 1,605 |  | 0,139 | 3,671 | 0,001* | 0,319 | 2,372 | 0,018* | 0,206 |
| 7 | 3,122 | 0,002* | 0,271 | 3,015 | 0,003* | 0,262 | 1,575 |  | 0,137 | 3,636 | 0,001* | 0,316 | 2,293 | 0,022 | 0,199 |
| 8 | 2,860 | 0,004* | 0,248 | 2,743 | 0,006* | 0,238 | 1,579 |  | 0,137 | 3,436 | 0,001* | 0,298 | 2,182 | 0,030 | 0,189 |
| 9 | 2,641 | 0,009* | 0,229 | 2,450 | 0,015* | 0,213 | 1,540 |  | 0,134 | 3,119 | 0,002* | 0,271 | 2,045 | 0,041 | 0,177 |
| 10 | 2,512 | 0,012* | 0,218 | 2,163 | 0,031 | 0,188 | 1,478 |  | 0,128 | 2,816 | 0,005* | 0,244 | 1,919 |  | 0,167 |
| 11 | 2,468 | 0,014* | 0,214 | 1,932 |  | 0,168 | 1,393 |  | 0,121 | 2,602 | 0,010* | 0,226 | 1,818 |  | 0,158 |
| 12 | 2,482 | 0,013* | 0,215 | 1,774 |  | 0,154 | 1,277 |  | 0,111 | 2,488 | 0,013* | 0,216 | 1,742 |  | 0,151 |

**Supplementary File 1x. Depth-dependent qT1 change per *a-priori* network *Perspective* vs *Affect*.** T-values and p-values below p<0.05, and Cohen’s D effect size, ***** indicates FDRp<0.05.

|  | Attention | | | Interoception | | | Emotion | | | Empathy | | | ToM | | |
| --- | --- | --- | --- | --- | --- | --- | --- | --- | --- | --- | --- | --- | --- | --- | --- |
| Depth | t | p | D | t- | p | D | t | p | D | t | p | D | t | p | D |
| 1 | -2,455 | 0,014 | -0,213 | -0,876 |  | -0,076 | 1,035 |  | 0,090 | -0,883 |  | -0,077 | -1,377 |  | -0,120 |
| 2 | -2,122 | 0,034 | -0,184 | -1,131 |  | -0,098 | 1,046 |  | 0,091 | -0,763 |  | -0,066 | -1,160 |  | -0,101 |
| 3 | -1,679 |  | -0,146 | -1,167 |  | -0,101 | 1,155 |  | 0,100 | -0,511 |  | -0,044 | -0,742 |  | -0,064 |
| 4 | -1,166 |  | -0,101 | -0,959 |  | -0,083 | 1,364 |  | 0,118 | -0,125 |  | -0,011 | -0,191 |  | -0,017 |
| 5 | -0,615 |  | -0,053 | -0,595 |  | -0,052 | 1,676 |  | 0,146 | 0,328 |  | 0,028 | 0,363 |  | 0,032 |
| 6 | -0,075 |  | -0,006 | -0,131 |  | -0,011 | 2,017 | 0,044 | 0,175 | 0,800 |  | 0,069 | 0,841 |  | 0,073 |
| 7 | 0,421 |  | 0,037 | 0,443 |  | 0,038 | 2,309 | 0,021 | 0,200 | 1,275 |  | 0,111 | 1,142 |  | 0,099 |
| 8 | 0,818 |  | 0,071 | 1,122 |  | 0,097 | 2,533 | 0,012* | 0,220 | 1,608 |  | 0,140 | 1,242 |  | 0,108 |
| 9 | 1,079 |  | 0,094 | 1,799 |  | 0,156 | 2,658 | 0,008* | 0,231 | 1,792 |  | 0,156 | 1,234 |  | 0,107 |
| 10 | 1,210 |  | 0,105 | 2,383 | 0,018* | 0,207 | 2,766 | 0,006* | 0,240 | 1,892 |  | 0,164 | 1,202 |  | 0,104 |
| 11 | 1,245 |  | 0,108 | 2,857 | 0,004* | 0,248 | 2,850 | 0,005* | 0,247 | 1,962 | 0,050 | 0,170 | 1,188 |  | 0,103 |
| 12 | 1,217 |  | 0,106 | 3,246 | 0,001* | 0,282 | 2,930 | 0,004* | 0,254 | 2,043 | 0,042 | 0,177 | 1,203 |  | 0,104 |

**Supplementary File 1y. Depth-dependent qT1 change per *a-priori* network *Presence* vs *Perspective* in TC1.** T-values and p-values below p<0.05. ***** indicates FDRp<0.05.

| Depth | Attention |  | Interoception | | Emotion |  | Empathy |  | ToM |  |
| --- | --- | --- | --- | --- | --- | --- | --- | --- | --- | --- |
| 1 | 4,136 | 0.001* | 2,618 | 0,010 | 0,568 |  | 3,285 | 0,001* | 3,445 | 0,001* |
| 2 | 3,741 | 0,001* | 2,873 | 0,005* | 0,421 |  | 3,228 | 0,002* | 2,951 | 0,004* |
| 3 | 3,165 | 0,002* | 2,857 | 0,005* | 0,144 |  | 2,922 | 0,004* | 2,152 | 0,033 |
| 4 | 2,553 | 0,012 | 2,599 | 0,010 | -0,166 |  | 2,544 | 0,012 | 1,390 |  |
| 5 | 1,984 | 0,049 | 2,244 | 0,026 | -0,413 |  | 2,254 | 0,026 | 0,897 |  |
| 6 | 1,522 |  | 1,900 |  | -0,535 |  | 2,102 | 0,037 | 0,709 |  |
| 7 | 1,188 |  | 1,556 |  | -0,561 |  | 1,996 | 0,048 | 0,757 |  |
| 8 | 0,983 |  | 1,225 |  | -0,545 |  | 1,871 |  | 0,908 |  |
| 9 | 0,891 |  | 0,883 |  | -0,554 |  | 1,696 |  | 1,019 |  |
| 10 | 0,881 |  | 0,547 |  | -0,601 |  | 1,543 |  | 1,063 |  |
| 11 | 0,910 |  | 0,236 |  | -0,688 |  | 1,425 |  | 1,048 |  |
| 12 | 0,941 |  | -0,061 |  | -0,868 |  | 1,314 |  | 0,964 |  |

**Supplementary File 1z. Depth-dependent qT1 change per *a-priori* network *Presence* vs *Affect* in TC1.** T-values and p-values below p<0.05. ***** indicates FDRp<0.05.

| Depth | Attention |  | Interoception | | Emotion |  | Empathy |  | ToM |  |
| --- | --- | --- | --- | --- | --- | --- | --- | --- | --- | --- |
| 1 | 3,483 | 0,001* | 2,057 | 0,041 | 0,757 |  | 2,975 | 0,003* | 2,837 | 0,005* |
| 2 | 3,765 | 0,000* | 2,584 | 0,011 | 0,626 |  | 3,330 | 0,001* | 2,687 | 0,008* |
| 3 | 3,756 | 0,000* | 3,005 | 0,003* | 0,464 |  | 3,464 | 0,001* | 2,296 | 0,023 |
| 4 | 3,544 | 0,001* | 3,250 | 0,001* | 0,291 |  | 3,439 | 0,001* | 1,821 | 0,071 |
| 5 | 3,226 | 0,002* | 3,326 | 0,001* | 0,156 |  | 3,369 | 0,001* | 1,434 |  |
| 6 | 2,860 | 0,005* | 3,262 | 0,001* | 0,041 |  | 3,278 | 0,001* | 1,189 |  |
| 7 | 2,484 | 0,014* | 3,080 | 0,002* | -0,034 |  | 3,048 | 0,003* | 1,008 |  |
| 8 | 2,126 | 0,035 | 2,811 | 0,006* | -0,083 |  | 2,637 | 0,009 | 0,843 |  |
| 9 | 1,836 | 0,068 | 2,466 | 0,015* | -0,136 |  | 2,159 | 0,032 | 0,696 |  |
| 10 | 1,671 | 0,097 | 2,145 | 0,034 | -0,161 |  | 1,797 | 0,074 | 0,585 |  |
| 11 | 1,619 |  | 1,898 |  | -0,206 |  | 1,571 |  | 0,474 |  |
| 12 | 1,651 |  | 1,737 |  | -0,320 |  | 1,444 |  | 0,354 |  |

**Supplementary File 1za. Depth-dependent qT1 change per *a-priori* network *Perspective* vs *Affect* in TC1.** T-values and p-values below p<0.05. ***** indicates FDRp<0.05.

| Depth | Attention |  | Interoception | | Emotion |  | Empathy |  | ToM |  |
| --- | --- | --- | --- | --- | --- | --- | --- | --- | --- | --- |
| 1 | -0,646 |  | -0,550 |  | 0,178 |  | -0,313 |  | -0,599 |  |
| 2 | 0,004 |  | -0,291 |  | 0,194 |  | 0,081 |  | -0,268 |  |
| 3 | 0,549 |  | 0,127 |  | 0,305 |  | 0,504 |  | 0,127 |  |
| 4 | 0,934 |  | 0,610 |  | 0,438 |  | 0,843 |  | 0,405 |  |
| 5 | 1,178 |  | 1,024 |  | 0,546 |  | 1,055 |  | 0,509 |  |
| 6 | 1,272 |  | 1,293 |  | 0,554 |  | 1,114 |  | 0,455 |  |
| 7 | 1,233 |  | 1,449 |  | 0,507 |  | 0,996 |  | 0,236 |  |
| 8 | 1,088 |  | 1,510 |  | 0,445 |  | 0,723 |  | -0,066 |  |
| 9 | 0,899 |  | 1,509 |  | 0,403 |  | 0,433 |  | -0,314 |  |
| 10 | 0,752 |  | 1,525 |  | 0,424 |  | 0,235 |  | -0,463 |  |
| 11 | 0,673 |  | 1,589 |  | 0,464 |  | 0,132 |  | -0,555 |  |
| 12 | 0,674 |  | 1,719 |  | 0,529 |  | 0,118 |  | -0,588 |  |

**Supplementary File 1zb. Depth-dependent qT1 change per *a-priori* network *Presence* vs *Perspective* in TC2.** T-values and p-values below p<0.05. ***** indicates FDRp<0.05.

| Depth | Attention |  | Interoception | | Emotion |  | Empathy |  | ToM |  |
| --- | --- | --- | --- | --- | --- | --- | --- | --- | --- | --- |
| 1 | 3,891 | 0,001* | 1,765 | 0,080 | 0,304 |  | 1,620 |  | 2,464 | 0,015 |
| 2 | 4,061 | 0,001* | 2,499 | 0,014 | 0,684 |  | 1,895 |  | 2,755 | 0,007 |
| 3 | 3,992 | 0,001* | 2,931 | 0,004 | 0,831 |  | 1,998 | 0,048 | 2,714 | 0,007 |
| 4 | 3,697 | 0,001* | 3,008 | 0,003 | 0,735 |  | 1,903 |  | 2,390 | 0,018 |
| 5 | 3,243 | 0,001 | 2,774 | 0,006 | 0,443 |  | 1,637 |  | 1,890 |  |
| 6 | 2,692 | 0,008 | 2,263 | 0,025 | 0,033 |  | 1,245 |  | 1,308 |  |
| 7 | 2,075 | 0,040 | 1,543 |  | -0,354 |  | 0,727 |  | 0,727 |  |
| 8 | 1,457 |  | 0,658 |  | -0,655 |  | 0,173 |  | 0,233 |  |
| 9 | 0,941 |  | -0,214 |  | -0,866 |  | -0,307 |  | -0,107 |  |
| 10 | 0,610 |  | -0,971 |  | -1,048 |  | -0,679 |  | -0,311 |  |
| 11 | 0,474 |  | -1,532 |  | -1,183 |  | -0,916 |  | -0,415 |  |
| 12 | 0,502 |  | -1,896 |  | -1,251 |  | -1,025 |  | -0,417 |  |

**Supplementary File 1zc. Depth-dependent qT1 change per *a-priori* network *Presence* vs *Affect* in TC2.** T-values and p-values below p<0.05. ***** indicates FDRp<0.05.

| Depth | Attention |  | Interoception | | Emotion |  | Empathy |  | ToM |  |
| --- | --- | --- | --- | --- | --- | --- | --- | --- | --- | --- |
| 1 | 2,851 | 0,005 | 2,071 | 0,040 | 1,006 |  | 1,617 |  | 2,005 | 0,047 |
| 2 | 2,871 | 0,005 | 2,501 | 0,013 | 1,468 |  | 1,887 |  | 2,424 | 0,017 |
| 3 | 2,783 | 0,006 | 2,668 | 0,008 | 1,746 |  | 2,078 | 0,039 | 2,650 | 0,009 |
| 4 | 2,621 | 0,010 | 2,593 | 0,010 | 1,906 |  | 2,197 | 0,030 | 2,771 | 0,006 |
| 5 | 2,436 | 0,016 | 2,343 | 0,021 | 2,017 | 0,046 | 2,281 | 0,024 | 2,840 | 0,005 |
| 6 | 2,243 | 0,026 | 2,002 | 0,047 | 2,063 | 0,041 | 2,354 | 0,020 | 2,860 | 0,005 |
| 7 | 2,032 | 0,044 | 1,637 |  | 2,061 | 0,041 | 2,353 | 0,020 | 2,758 | 0,007 |
| 8 | 1,793 |  | 1,308 |  | 2,016 | 0,046 | 2,235 | 0,027 | 2,514 | 0,013 |
| 9 | 1,563 |  | 1,046 |  | 1,925 |  | 2,036 | 0,044 | 2,233 | 0,027 |
| 10 | 1,376 |  | 0,813 |  | 1,767 |  | 1,800 |  | 1,976 |  |
| 11 | 1,252 |  | 0,657 |  | 1,579 |  | 1,604 |  | 1,778 |  |
| 12 | 1,192 |  | 0,560 |  | 1,391 |  | 1,484 |  | 1,632 |  |

**Supplementary File 1zd. Depth-dependent qT1 change per *a-priori* network *Perspective* vs *Affect* in TC2.** T-values and p-values below p<0.05. ***** indicates FDRp<0.05.

| Depth | Attention |  | Interoception | | Emotion |  | Empathy |  | ToM |  |
| --- | --- | --- | --- | --- | --- | --- | --- | --- | --- | --- |
| 1 | -1,156 |  | 0,240 |  | 0,680 |  | -0,059 |  | -0,536 |  |
| 2 | -1,312 |  | -0,085 |  | 0,747 |  | -0,074 |  | -0,420 |  |
| 3 | -1,327 |  | -0,361 |  | 0,870 |  | 0,011 |  | -0,157 |  |
| 4 | -1,187 |  | -0,513 |  | 1,125 |  | 0,223 |  | 0,291 |  |
| 5 | -0,907 |  | -0,521 |  | 1,532 |  | 0,576 |  | 0,868 |  |
| 6 | -0,535 |  | -0,336 |  | 1,993 | 0,048 | 1,047 |  | 1,480 |  |
| 7 | -0,114 |  | 0,039 |  | 2,385 | 0,018 | 1,572 |  | 1,971 |  |
| 8 | 0,280 |  | 0,616 |  | 2,647 | 0,009 | 2,020 | 0,045 | 2,234 | 0,027 |
| 9 | 0,578 |  | 1,245 |  | 2,772 | 0,006 | 2,312 | 0,022 | 2,303 | 0,023 |
| 10 | 0,731 |  | 1,787 |  | 2,801 | 0,006 | 2,459 | 0,015 | 2,258 | 0,025 |
| 11 | 0,748 |  | 2,204 | 0,029 | 2,754 | 0,007 | 2,508 | 0,013 | 2,168 | 0,032 |
| 12 | 0,661 |  | 2,479 | 0,014 | 2,640 | 0,009 | 2,501 | 0,013 | 2,028 | 0,044 |

**Supplementary File 1ze. Depth-dependent qT1 change per *a-priori* network baseline – T1: TC1 (*Presence*) versus Retest Control.** T-values and p-values below p<0.05. ***** indicates FDRp<0.05.

| Depth | Attention | | Interoception | | Emotion |  | Empathy |  | ToM |  |
| --- | --- | --- | --- | --- | --- | --- | --- | --- | --- | --- |
| 1 | -0,188 |  | 0,236 |  | 0,691 |  | 0,296 |  | -0,002 |  |
| 2 | -0,484 |  | 0,082 |  | 0,907 |  | 0,379 |  | -0,316 |  |
| 3 | -0,764 |  | -0,159 |  | 0,908 |  | 0,286 |  | -0,649 |  |
| 4 | -1,049 |  | -0,368 |  | 0,691 |  | 0,152 |  | -0,968 |  |
| 5 | -1,268 |  | -0,507 |  | 0,339 |  | 0,000 |  | -1,259 |  |
| 6 | -1,401 |  | -0,623 |  | -0,066 |  | -0,132 |  | -1,465 |  |
| 7 | -1,429 |  | -0,625 |  | -0,409 |  | -0,223 |  | -1,507 |  |
| 8 | -1,334 |  | -0,530 |  | -0,624 |  | -0,266 |  | -1,348 |  |
| 9 | -1,152 |  | -0,368 |  | -0,705 |  | -0,306 |  | -1,066 |  |
| 10 | -0,966 |  | -0,158 |  | -0,684 |  | -0,253 |  | -0,783 |  |
| 11 | -0,847 |  | 0,053 |  | -0,600 |  | -0,143 |  | -0,525 |  |
| 12 | -0,820 |  | 0,206 |  | -0,454 |  | 0,006 |  | -0,276 |  |

**Supplementary File 1zf. Depth-dependent qT1 change per *a-priori* network baseline – T1: TC2 (*Presence*) versus Retest Control.** T-values and p-values below p<0.05. ***** indicates FDRp<0.05.

| Depth | Attention | | Interoception | | Emotion |  | Empathy |  | ToM |  |
| --- | --- | --- | --- | --- | --- | --- | --- | --- | --- | --- |
| 1 | -0,371 |  | -0,052 |  | 0,467 |  | -0,825 |  | -0,286 |  |
| 2 | -0,422 |  | 0,209 |  | 1,220 |  | -0,474 |  | 0,119 |  |
| 3 | -0,487 |  | 0,257 |  | 1,672 |  | -0,259 |  | 0,409 |  |
| 4 | -0,634 |  | 0,133 |  | 1,766 |  | -0,139 |  | 0,475 |  |
| 5 | -0,783 |  | -0,094 |  | 1,567 |  | -0,167 |  | 0,327 |  |
| 6 | -0,935 |  | -0,441 |  | 1,132 |  | -0,324 |  | 0,040 |  |
| 7 | -1,093 |  | -0,744 |  | 0,605 |  | -0,577 |  | -0,297 |  |
| 8 | -1,231 |  | -0,985 |  | 0,132 |  | -0,870 |  | -0,578 |  |
| 9 | -1,319 |  | -1,089 |  | -0,196 |  | -1,145 |  | -0,721 |  |
| 10 | -1,373 |  | -1,049 |  | -0,393 |  | -1,307 |  | -0,758 |  |
| 11 | -1,409 |  | -0,885 |  | -0,454 |  | -1,347 |  | -0,686 |  |
| 12 | -1,439 |  | -0,667 |  | -0,341 |  | -1,264 |  | -0,486 |  |

**Supplementary File 1zg. Depth-dependent qT1 change per *a-priori* network baseline – T1: *Affect* TC3 vs Retest Control.** T-values and p-values below p<0.05. ***** indicates FDRp<0.05.

| Depth | Attention | | Interoception | | Emotion |  | Empathy |  | ToM |  |
| --- | --- | --- | --- | --- | --- | --- | --- | --- | --- | --- |
| 1 | -1,397 |  | -0,818 |  | -0,896 |  | -1,493 |  | -1,420 |  |
| 2 | -1,728 |  | -0,881 |  | -0,525 |  | -1,278 |  | -1,382 |  |
| 3 | -1,948 |  | -0,987 |  | -0,209 |  | -1,031 |  | -1,278 |  |
| 4 | -2,089 | 0,038 | -1,093 |  | -0,078 |  | -0,903 |  | -1,218 |  |
| 5 | -2,205 | 0,029 | -1,195 |  | -0,178 |  | -0,968 |  | -1,303 |  |
| 6 | -2,341 | 0,020 | -1,284 |  | -0,522 |  | -1,227 |  | -1,513 |  |
| 7 | -2,488 | 0,013 | -1,383 |  | -0,965 |  | -1,614 |  | -1,744 |  |
| 8 | -2,628 | 0,009 | -1,471 |  | -1,344 |  | -1,957 |  | -1,863 |  |
| 9 | -2,734 | 0,007 | -1,504 |  | -1,578 |  | -2,134 | 0,034 | -1,835 |  |
| 10 | -2,821 | 0,005 | -1,446 |  | -1,688 |  | -2,175 | 0,031 | -1,751 |  |
| 11 | -2,904 | 0,004 | -1,317 |  | -1,701 |  | -2,157 | 0,032 | -1,665 |  |
| 12 | -2,978 | 0,003 | -1,155 |  | -1,618 |  | -2,118 | 0,035 | -1,578 |  |

**Supplementary File 1zh. Depth-dependent qT1 change per *a-priori* network baseline – T1: *Presence* TC1 vs *Affect* TC3.** T-values and p-values below p<0.05. ***** indicates FDRp<0.05.

| Depth | Attention | | Interoception | | Emotion |  | Empathy |  | ToM |  |
| --- | --- | --- | --- | --- | --- | --- | --- | --- | --- | --- |
| 1 | 1,234 |  | 1,078 |  | 1,624 |  | 1,829 |  | 1,449 |  |
| 2 | 1,269 |  | 0,984 |  | 1,467 |  | 1,684 |  | 1,088 |  |
| 3 | 1,207 |  | 0,845 |  | 1,145 |  | 1,326 |  | 0,640 |  |
| 4 | 1,047 |  | 0,740 |  | 0,788 |  | 1,059 |  | 0,253 |  |
| 5 | 0,934 |  | 0,698 |  | 0,529 |  | 0,968 |  | 0,041 |  |
| 6 | 0,932 |  | 0,661 |  | 0,465 |  | 1,097 |  | 0,044 |  |
| 7 | 1,054 |  | 0,755 |  | 0,567 |  | 1,405 |  | 0,237 |  |
| 8 | 1,297 |  | 0,939 |  | 0,734 |  | 1,726 |  | 0,522 |  |
| 9 | 1,597 |  | 1,140 |  | 0,890 |  | 1,867 |  | 0,782 |  |
| 10 | 1,877 |  | 1,301 |  | 1,024 |  | 1,963 |  | 0,987 |  |
| 11 | 2,077 | 0,039 | 1,389 |  | 1,123 |  | 2,057 | 0,041 | 1,163 |  |
| 12 | 2,166 | 0,031 | 1,382 |  | 1,187 |  | 2,171 | 0,031 | 1,329 |  |

**Supplementary File 1zi. Depth-dependent qT1 change per *a-priori* network baseline – T1: *Presence* TC2 vs *Affect* TC3.** T-values and p-values below p<0.05. ***** indicates FDRp<0.05.

| Depth | Attention | | Interoception | | Emotion |  | Empathy |  | ToM |  |
| --- | --- | --- | --- | --- | --- | --- | --- | --- | --- | --- |
| 1 | 0,988 |  | 0,748 |  | 1,356 |  | 0,616 |  | 1,097 |  |
| 2 | 1,259 |  | 1,077 |  | 1,764 |  | 0,757 |  | 1,475 |  |
| 3 | 1,408 |  | 1,230 |  | 1,918 |  | 0,725 |  | 1,670 |  |
| 4 | 1,381 |  | 1,207 |  | 1,886 |  | 0,722 |  | 1,680 |  |
| 5 | 1,332 |  | 1,070 |  | 1,779 |  | 0,756 |  | 1,611 |  |
| 6 | 1,306 |  | 0,790 |  | 1,670 |  | 0,848 |  | 1,522 |  |
| 7 | 1,288 |  | 0,573 |  | 1,565 |  | 0,973 |  | 1,404 |  |
| 8 | 1,288 |  | 0,412 |  | 1,452 |  | 1,024 |  | 1,231 |  |
| 9 | 1,308 |  | 0,340 |  | 1,344 |  | 0,916 |  | 1,057 |  |
| 10 | 1,340 |  | 0,329 |  | 1,250 |  | 0,791 |  | 0,938 |  |
| 11 | 1,377 |  | 0,374 |  | 1,200 |  | 0,732 |  | 0,927 |  |
| 12 | 1,405 |  | 0,440 |  | 1,235 |  | 0,778 |  | 1,047 |  |

**Supplementary File 1zj. Depth-dependent qT1 change per *a-priori* network T1 – T3: *Perspective* versus *Affect* (TC1+TC2).** T-values and p-values below p<0.05. ***** indicates FDRp<0.05.

| Affect-Control  Depth | Attention | | Interoception | | Emotion |  | Empathy |  | ToM |  |
| --- | --- | --- | --- | --- | --- | --- | --- | --- | --- | --- |
| 1 | -1,175 |  | -0,205 |  | 0,659 |  | -0,202 |  | -0,749 |  |
| 2 | -0,862 |  | -0,236 |  | 0,720 |  | 0,061 |  | -0,444 |  |
| 3 | -0,499 |  | -0,133 |  | 0,904 |  | 0,430 |  | 0,004 |  |
| 4 | -0,112 |  | 0,105 |  | 1,205 |  | 0,867 |  | 0,534 |  |
| 5 | 0,283 |  | 0,410 |  | 1,598 |  | 1,328 |  | 1,043 |  |
| 6 | 0,631 |  | 0,754 |  | 1,934 |  | 1,734 |  | 1,434 |  |
| 7 | 0,901 |  | 1,151 |  | 2,149 | 0,032 | 2,005 |  | 1,592 |  |
| 8 | 1,057 |  | 1,619 |  | 2,246 | 0,025 | 2,086 | 0,046 | 1,537 |  |
| 9 | 1,105 |  | 2,080 | 0,038 | 2,270 | 0,024 | 2,051 | 0,038 | 1,401 |  |
| 10 | 1,084 |  | 2,482 | 0,014 | 2,293 | 0,023 | 1,988 | 0,041 | 1,266 |  |
| 11 | 1,030 |  | 2,808 | 0,005 | 2,294 | 0,022 | 1,930 |  | 1,143 |  |
| 12 | 0,968 |  | 3,043 | 0,003 | 2,275 | 0,024 | 1,900 |  | 1,036 |  |

**Supplementary File 1zk. Depth-dependent qT1 change per *a-priori* network T1 – T3: *Affect* vs Retest Control. *** T-values and p-values below p<0.05. ***** indicates FDRp<0.05.

| Affect-Control  Depth | Attention | | Interoception | | Emotion |  | Empathy |  | ToM |  |
| --- | --- | --- | --- | --- | --- | --- | --- | --- | --- | --- |
| 1 | 0,237 |  | 0,124 |  | -0,569 |  | -0,078 |  | -0,030 |  |
| 2 | -0,059 |  | 0,105 |  | -1,025 |  | -0,517 |  | -0,495 |  |
| 3 | -0,363 |  | -0,028 |  | -1,459 |  | -1,010 |  | -0,926 |  |
| 4 | -0,633 |  | -0,242 |  | -1,798 |  | -1,446 |  | -1,305 |  |
| 5 | -0,858 |  | -0,473 |  | -2,004 | 0,046 | -1,756 |  | -1,544 |  |
| 6 | -1,007 |  | -0,681 |  | -2,015 | 0,045 | -1,907 |  | -1,624 |  |
| 7 | -1,072 |  | -0,867 |  | -1,890 |  | -1,871 |  | -1,555 |  |
| 8 | -1,062 |  | -1,068 |  | -1,735 |  | -1,701 |  | -1,387 |  |
| 9 | -1,025 |  | -1,279 |  | -1,614 |  | -1,512 |  | -1,238 |  |
| 10 | -1,000 |  | -1,483 |  | -1,563 |  | -1,389 |  | -1,136 |  |
| 11 | -1,007 |  | -1,678 |  | -1,563 |  | -1,344 |  | -1,073 |  |
| 12 | -1,047 |  | -1,858 |  | -1,629 |  | -1,392 |  | -1,084 |  |

**Supplementary File 1zl. Depth-dependent qT1 change per *a-priori* network T1 – T3: *Perspective* vs Retest Control.** T-values and p-values below p<0.05. ***** indicates FDRp<0.05.

| Depth | Attention | | Interoception | | Emotion |  | Empathy |  | ToM |  |
| --- | --- | --- | --- | --- | --- | --- | --- | --- | --- | --- |
| 1 | -0,937 |  | -0,088 |  | 0,09 |  | -0,276 |  | -0,794 |  |
| 2 | -0,908 |  | -0,132 |  | -0,293 |  | -0,446 |  | -0,933 |  |
| 3 | -0,845 |  | -0,159 |  | -0,535 |  | -0,565 |  | -0,903 |  |
| 4 | -0,729 |  | -0,134 |  | -0,569 |  | -0,561 |  | -0,743 |  |
| 5 | -0,562 |  | -0,061 |  | -0,382 |  | -0,412 |  | -0,471 |  |
| 6 | -0,368 |  | 0,071 |  | -0,062 |  | -0,162 |  | -0,162 |  |
| 7 | -0,168 |  | 0,276 |  | 0,273 |  | 0,138 |  | 0,066 |  |
| 8 | -0,005 |  | 0,537 |  | 0,522 |  | 0,385 |  | 0,183 |  |
| 9 | 0,079 |  | 0,784 |  | 0,665 |  | 0,536 |  | 0,202 |  |
| 10 | 0,084 |  | 0,982 |  | 0,738 |  | 0,597 |  | 0,172 |  |
| 11 | 0,025 |  | 1,117 |  | 0,736 |  | 0,583 |  | 0,110 |  |
| 12 | -0,074 |  | 1,183 |  | 0,649 |  | 0,505 |  | -0,011 |  |

**Supplementary File 1zm. Depth-dependent qT1 change per *a-priori* network controlling for CTX: *Presence* versus *Perspective*.** T-values and p-values below p<0.05. ***** indicates FDRp<0.05.

| Depth | Attention |  | Interoception | | Emotion |  | Empathy |  | ToM |  |
| --- | --- | --- | --- | --- | --- | --- | --- | --- | --- | --- |
| 1 | 5,664 | 0,001* | 3,264 | 0,001* | 0,425 |  | 3,468 | 0,001* | 3,897 | 0,001* |
| 2 | 5,568 | 0,001* | 3,939 | 0,001* | 0,528 |  | 3,655 | 0,001* | 3,788 | 0,001* |
| 3 | 5,155 | 0,001* | 4,205 | 0,001* | 0,396 |  | 3,568 | 0,001* | 3,278 | 0,001* |
| 4 | 4,520 | 0,001* | 4,049 | 0,001* | 0,082 |  | 3,249 | 0,001* | 2,524 | 0,012* |
| 5 | 3,768 | 0,001* | 3,602 | 0,001* | -0,337 |  | 2,821 | 0,005* | 1,767 |  |
| 6 | 2,996 | 0,003* | 2,962 | 0,003* | -0,759 |  | 2,377 | 0,018* | 1,163 |  |
| 7 | 2,261 | 0,024 | 2,164 | 0,031 | -1,089 |  | 1,881 |  | 0,766 |  |
| 8 | 1,634 |  | 1,240 |  | -1,296 |  | 1,376 |  | 0,544 |  |
| 9 | 1,196 |  | 0,313 |  | -1,437 |  | 0,924 |  | 0,423 |  |
| 10 | 0,984 |  | -0,493 |  | -1,574 |  | 0,577 |  | 0,354 |  |
| 11 | 0,959 |  | -1,128 |  | -1,700 |  | 0,344 |  | 0,308 |  |
| 12 | 1,068 |  | -1,615 |  | -1,833 |  | 0,196 |  | 0,276 |  |

**Supplementary File 1zn. Depth-dependent qT1 change per *a-priori* network controlling for CTX: *Presence* versus *Affect*.** T-values and p-values below p<0.05. ***** indicates FDRp<0.05.

| Depth | Attention |  | Interoception | | Emotion |  | Empathy |  | ToM |  |
| --- | --- | --- | --- | --- | --- | --- | --- | --- | --- | --- |
| 1 | 3,914 | 0,001* | 2,793 | 0,005* | 1,372 |  | 3,114 | 0,002* | 2,885 | 0,004* |
| 2 | 4,140 | 0,001* | 3,274 | 0,001* | 1,534 |  | 3,459 | 0,001* | 3,006 | 0,003* |
| 3 | 4,125 | 0,001* | 3,544 | 0,001* | 1,524 |  | 3,654 | 0,001* | 2,908 | 0,004* |
| 4 | 3,938 | 0,001* | 3,612 | 0,001* | 1,390 |  | 3,716 | 0,001* | 2,680 | 0,008* |
| 5 | 3,669 | 0,001* | 3,540 | 0,001* | 1,230 |  | 3,738 | 0,001* | 2,464 | 0,014* |
| 6 | 3,379 | 0,001* | 3,385 | 0,001* | 1,084 |  | 3,770 | 0,001* | 2,342 | 0,020 |
| 7 | 3,086 | 0,002* | 3,190 | 0,002* | 0,985 |  | 3,704 | 0,001* | 2,243 | 0,025 |
| 8 | 2,806 | 0,005* | 2,959 | 0,003* | 0,916 |  | 3,478 | 0,001* | 2,101 | 0,036 |
| 9 | 2,582 | 0,010* | 2,683 | 0,008* | 0,858 |  | 3,135 | 0,002* | 1,936 |  |
| 10 | 2,463 | 0,014* | 2,388 | 0,017* | 0,806 |  | 2,810 | 0,005* | 1,790 |  |
| 11 | 2,444 | 0,015* | 2,137 | 0,033 | 0,747 |  | 2,574 | 0,010* | 1,678 |  |
| 12 | 2,498 | 0,013* | 1,927 |  | 0,682 |  | 2,439 | 0,015* | 1,602 |  |

**Supplementary File 1zo. Depth-dependent qT1 change per *a-priori* network controlling for CTX: *Perspective* versus *Affect*.** T-values and p-values below p<0.05. ***** indicates FDRp<0.05.

| Depth | Attention |  | Interoception | | Emotion |  | Empathy |  | ToM |  |
| --- | --- | --- | --- | --- | --- | --- | --- | --- | --- | --- |
| 1 | -2,386 | 0,017* | -0,859 |  | 0,857 |  | -0,776 |  | -1,461 |  |
| 2 | -2,066 | 0,039 | -1,127 |  | 0,901 |  | -0,650 |  | -1,227 |  |
| 3 | -1,632 |  | -1,157 |  | 1,035 |  | -0,371 |  | -0,770 |  |
| 4 | -1,122 |  | -0,922 |  | 1,247 |  | 0,037 |  | -0,170 |  |
| 5 | -0,564 |  | -0,506 |  | 1,549 |  | 0,524 |  | 0,446 |  |
| 6 | -0,006 |  | 0,040 |  | 1,869 |  | 1,038 |  | 0,987 |  |
| 7 | 0,511 |  | 0,717 |  | 2,133 | 0,033 | 1,514 |  | 1,324 |  |
| 8 | 0,923 |  | 1,497 |  | 2,292 | 0,022 | 1,848 |  | 1,428 |  |
| 9 | 1,182 |  | 2,238 | 0,026 | 2,388 | 0,017* | 2,009 | 0,045 | 1,401 |  |
| 10 | 1,298 |  | 2,831 | 0,005* | 2,486 | 0,013* | 2,074 | 0,039 | 1,336 |  |
| 11 | 1,306 |  | 3,283 | 0,001* | 2,564 | 0,011* | 2,100 | 0,036 | 1,278 |  |
| 12 | 1,240 |  | 3,612 | 0,001* | 2,646 | 0,008* | 2,131 | 0,034 | 1,240 |  |
